# A novel viral regulatory network for autophagy induction: Respiratory Syncytial Virus NS2 protein regulates autophagy by modulating BECN1 ISGylation and protein stability

**DOI:** 10.1080/27694127.2022.2076769

**Published:** 2022-05-16

**Authors:** Kim Chiok, Santanu Bose

**Affiliations:** Department of Veterinary Microbiology and Pathology, College of Veterinary Medicine, Washington State University, Pullman, Washington, USA

**Keywords:** Autophagy, Beclin1, dysregulation, ISGylation, NS2, proteasomal degradation, respiratory syncytial virus

## Abstract

Respiratory syncytial virus, or RSV, is a leading cause of viral pneumonia and bronchiolitis in children and other susceptible populations. RSV infection dysregulates the immune response leading to exaggerated inflammation in the airway. Among other responses, RSV induces macroautophagy/autophagy, a key process that regulates immune response during infection. We investigated the molecular mechanisms underlying RSV-induced autophagy and showed that the RSV nonstructural NS2 protein promotes autophagy using a dual mechanism. First, NS2 interacts with and stabilizes the autophagy regulator BECN1 (beclin 1), augmenting its intracellular availability for autophagy induction. Second, NS2 interferes with BECN1 ISGylation, thus restricting the intracellular pool of the anti-autophagy ISGylated form of BECN1. Thus, the viral protein (i.e., NS2)-autophagy-ISGylation axis represents a yet unknown regulatory network for viruses. As many viruses induce autophagy that shapes virus-associated immune responses including inflammation, exploring viral protein-autophagy-ISGylation regulatory networks can aid in developing interventions to curb exaggerated immune responses such as inflammation for treating virus-associated inflammatory diseases.

Autophagy is a cellular process that preserves cell viability under unfavorable conditions by degrading and recycling cellular components through the use of compartments known as autophagosomes. During virus infection, autophagy regulates multiple arms of the virus-associated immune response. Autophagy is also a regulatory arm of the immune response that promotes immune cell differentiation, cell signaling, and cytokine release, among others. Many viruses manipulate autophagy to facilitate their spread, including human respiratory syncytial virus (RSV). RSV causes pneumonia and bronchiolitis in infants, elder patients and the immunocompromised. Vaccines are unavailable and limited therapeutic options result in significant mortality and morbidity. RSV infection induces autophagy in lung cells, dendritic cells and macrophages via unknown mechanisms. We recently investigated how RSV induces autophagy in light of its role in shaping infection dynamics and immune response.

In a recent paper [1], we established how the RSV encoded protein NS2 promotes autophagy by a dual mechanism: stabilizing the autophagy mediator BECN1 and then downregulating its ISGylation to restrict the formation of anti-autophagy ISGylated-BECN1. NS2 is a nonstructural protein of RSV that is synthesized in infected cells but is not packaged into newly assembled viruses. Western blot analysis shows that NS2 induces autophagy as its expression increases protein levels of the autophagy marker LC3-II, the lipidated form of LC3-I that is integrated into phagophores. Fluorescence microscopy confirms enhanced formation of LC3-II puncta in lung epithelial cells, indicating that enriched LC3-II correlates with subsequent incorporation into phagophores. In the presence of the autophagic flux inhibitor BafA1, we demonstrated that NS2 does not block autophagy function that may account for enhanced LC3-II or LC3-II puncta formation. To dissect NS2-mediated induction of autophagy, we examined the relationship between BECN1 and NS2 due to the former’s role as an autophagy regulator and the latter’s propensity to interact with cellular proteins. Immunoprecipitation assays showed that NS2 coprecipitates with BECN1, indicating the existence of an NS2-BECN1 complex that is further confirmed by colocalization assays. The NS2-BECN1 complex develops through interaction of NS2 with multiple domains of BECN1. Therefore, a nonstructural viral protein like NS2 induces autophagy *de novo*, a novel function characterized by the formation of an NS2-BECN1 complex.

To understand the implications of the NS2-BECN1 complex, we monitored BECN1 levels in the presence of NS2. BECN1 is enhanced in cells expressing NS2, an event that occurs post-translationally because *becn1* transcript levels remain unchanged. Experiments using protein synthesis and proteasomal inhibitors show that NS2 enhances BECN1 stability by slowing its degradation. During infection of lung epithelial cells, RSV induces autophagy as shown by conversion of nonlipidated LC3-I to lipidated LC3-II and degradation of the autophagy flux marker SQSTM1/p62. RSV infection also induces enhancement of BECN1 levels, linking BECN1 stabilization to functional autophagy, similar to the results obtained with non-infected cells expressing NS2. Thus, NS2 stabilizes BECN1 to ensure that an abundant pool of BECN1 is available for autophagy. Our studies with wild-type RSV and RSV lacking NS2 demonstrate that NS2 contributes to RSV-dependent autophagy via its association with BECN1 as RSV lacking NS2 fails to stabilize BECN1 and induce autophagy.

BECN1 interacts with members of the autophagy core complex to form the class III phosphatidylinositol 3-kinase complex that drives autophagy initiation and maturation. Two post-translational modifications on BECN1 govern the accessibility of its binding sites to interact with autophagy core complex proteins for autophagy induction. Conjugation of BECN1 to ubiquitin and ISG15 (ISG15 ubiquitin like modifier) results in BECN1 ubiquitination and ISGylation, respectively. Polyubiquitination at Lys63 enables interaction of BECN1 with autophagy partners and, thus, ubiquitination of BECN1 is a proautophagy modification. In contrast, covalent conjugation of ISG15 to BECN1, or ISGylation, at Lys117, Lys263, Lys265, and Lys266 competes with Lys63 polyubiquitination and, thus, BECN1 ISGylation is an anti-autophagy modification. Both BECN1 ubiquitination and ISGylation act as a regulatory system to “fine tune” autophagy by having opposite effects. We show that RSV NS2 protein disrupts this regulatory system by impairing ISGylation of BECN1. Using IFNB to stimulate ISGylation, our study shows that expression of NS2 reduces the formation of the anti-autophagy ISGylated BECN1. RSV NS2 therefore promotes autophagy by two mechanisms – a) NS2 increases the pool of available BECN1 for autophagy induction by preventing its degradation (Figure 1), and b) NS2 inhibits formation of the anti-autophagy ISGylated BECN1 by reducing BECN1 ISGylation (Figure 1).

**Figure 1.**
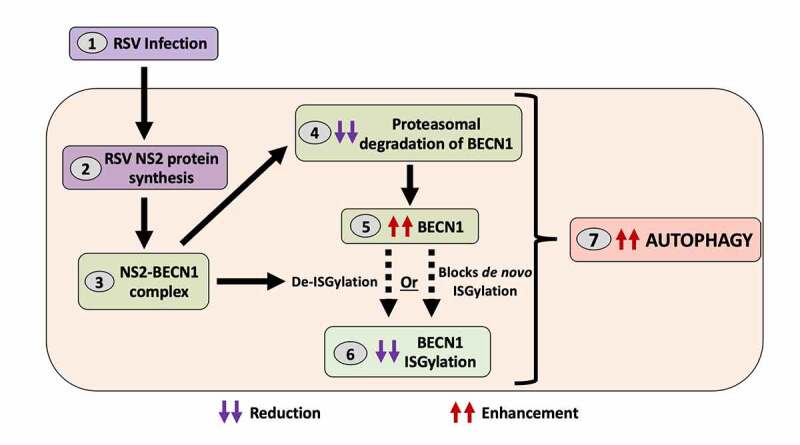
Respiratory syncytial virus (RSV) NS2 protein regulates autophagy by modulating BECN1 stability and ISGylation. After infection with RSV (1), the viral NS2 protein (2) forms a complex with the autophagy mediator BECN1 (3) and prevents its proteasomal degradation (4), increasing BECN1 abundance (5). NS2 also dampens the formation of anti-autophagy ISGylated BECN1 (6) to promote the pro-autophagy activity of BECN1 (7). It is unknown whether NS2 reduces BECN1 ISGylation via de-ISGylation or by blocking *de novo* ISGylation (dashed arrows).

RSV belongs to the *paramyxoviridae* family of RNA viruses. Highly pathogenic paramyxoviruses include RSV, parainfluenza viruses, measles, Nipah and Hendra viruses. All paramyxoviruses encode non-structural proteins that regulate the innate immune and inflammatory responses. However, the ability of paramyxovirus proteins in autophagy regulation had not been reported previously nor had a link between a viral protein, autophagy and ISGylation been established. Our study shows the ability of RSV NS2 protein to modulate ISGylation of BECN1 to trigger autophagy. The immunoregulatory activity of autophagy plays a key role during respiratory virus infection as these viruses provoke dysregulated immune responses, including exaggerated lung inflammation leading to pneumonia. Understanding the mechanisms governing immune dysregulation during respiratory virus infection is essential for developing schemes to prevent and treat pneumonias of viral origin. Our study with RSV, a clinically important respiratory virus, sheds light on unexplored viral protein-autophagy-ISGylation networks that could be targeted to develop therapeutics for virus-associated lung diseases.

The early synthesis of nonstructural proteins (e.g., NS2) during infection is perhaps an evolutionary advantage that ensures speedy control of the immune response to safeguard seizure of the cell’s resources. It is unknown whether NS2 reduces BECN1 ISGylation by de-ISGylating preformed ISGylated BECN1 or by blocking *de novo* conjugation of ISG15 to BECN1. Other viruses remain unexplored in their ability to direct viral protein-autophagy-ISGylation regulatory networks though many viruses, including influenza and coronaviruses, target ISGylation and autophagy pathways individually. Our study uncovered a novel viral mechanism wherein a viral protein controls ISGylation to modulate autophagy, a central branch of the immune response. As the first example of its kind, we show that the RSV NS2 stabilizes the autophagy regulator BECN1 and makes it available for autophagy by disrupting BECN1 ISGylation, promoting RSV-dependent autophagy.
